# Analytical modelling of monolayer graphene-based ion-sensitive FET to pH changes

**DOI:** 10.1186/1556-276X-8-173

**Published:** 2013-04-16

**Authors:** Mohammad Javad Kiani, Mohammad Taghi Ahmadi, Hediyeh Karimi Feiz Abadi, Meisam Rahmani, Amin Hashim, Fauzan Khairi Che harun

**Affiliations:** 1 Faculty of Electrical Engineering, Universiti Teknologi Malaysia, Skudai, Johor Bahru, 81310, Malaysia; 2 Department of Electrical Engineering, Islamic Azad University, Yasooj Branch, Yasooj, 7591483587, Iran; 3 Malaysia-Japan International Institute of Technology (MJIIT), Universiti Teknologi Malaysia, Johor Bahru, 54100, Malaysia; 4 Centre for Artificial Intelligence and Robotics (CAIRO), Faculty of Electrical Engineering, Universiti Teknologi Malaysia, Johor Bahru, 81310, Malaysia

**Keywords:** Graphene, Ion-sensitive field-effect transistor (ISFET), pH sensor, Conductance

## Abstract

Graphene has attracted great interest because of unique properties such as high sensitivity, high mobility, and biocompatibility. It is also known as a superior candidate for pH sensing. Graphene-based ion-sensitive field-effect transistor (ISFET) is currently getting much attention as a novel material with organic nature and ionic liquid gate that is intrinsically sensitive to pH changes. pH is an important factor in enzyme stabilities which can affect the enzymatic reaction and broaden the number of enzyme applications. More accurate and consistent results of enzymes must be optimized to realize their full potential as catalysts accordingly. In this paper, a monolayer graphene-based ISFET pH sensor is studied by simulating its electrical measurement of buffer solutions for different pH values. Electrical detection model of each pH value is suggested by conductance modelling of monolayer graphene. Hydrogen ion (H^+^) concentration as a function of carrier concentration is proposed, and the control parameter (*Ƥ*) is defined based on the electro-active ions absorbed by the surface of the graphene with different pH values. Finally, the proposed new analytical model is compared with experimental data and shows good overall agreement.

## Background

**Figure 1:**
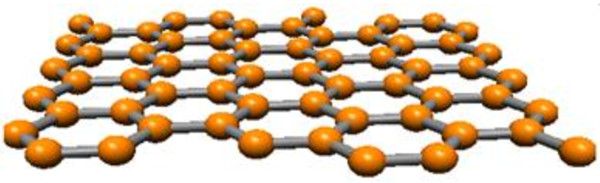
Monolayer graphene atom arrangement with only one atom thickness.

Graphene has two *sp*^2^-bonded carbon atoms, which make its structure apparently look like a honeycomb crystal as seen in Figure 1[1-3]. Because of its unique properties, graphene has attracted huge interest mainly in the electrical, physical, chemical, and even biological fields [4,5].

**Figure 2:**
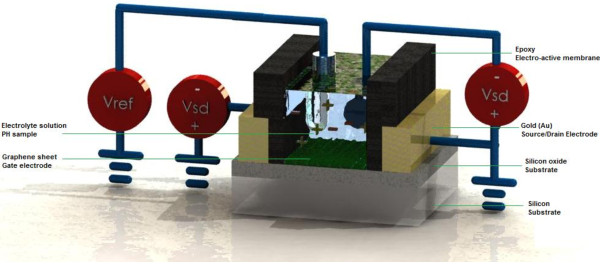
Schematic of the proposed structure and the electrical circuit of graphene based-ISFET for pH detection.

Nowadays, ion-sensitive field-effect transistors (ISFETs) have caught much attention due to their advantages such as small size and the possibilities for mass production [6,7]. Their short and consistent response times are very favorable to the electronics industry [8,9]. ISFETs introduce new features such as the integration of data processing and compensation circuits in the similar circuit for this type of sensors [10-12]. By altering the gate material, depositing layers of selective membrane or a bio-recognition element onto the gate, variance of selectivity can be achieved [13,14]. After the process of depositing, the sensors are now called chemically sensitive FETs [15,16]. Initially, heterogeneous membranes of silver halides and membranes based on polyvinyl chloride (PVC) have been used for ISFET [17,18]. Due to poor adherence between PVC base membrane and ISFET surface and inconsistent results, scientists explore for a new type of membrane [18,19]. That is where photocured polymers, which are compatible with the proposed photolithography techniques, come in [19,20]. They have the properties of a higher adherence string of the salinized ISFET gate's surface [21]. In order to expand ion-selective membranes, numerous polymers such as polysiloxanes, polyurethanes, and different methacrylate-derived polymers have been reported to be good candidates [22,23]. These new polymers show promising results regarding consistency and longer stability compared to PVC membranes [24]. In addition, almost all effective ion-based ISFETs were developed for clinical analyses and environmental applications [24]. Recently, microelectronic advances have been exploited and applied to improve ISFET fabrication methods [25,26]. Because of the electrolyte's ionic properties, electrical parts of ISFETs cannot have contact with liquid and only the gate area is open [27]. Due to its organic nature, the gate material for ISFETs is intrinsically sensitive to pH changes [28,29]. On the other hand, all enzymes are sensitive to pH changes, but extremely high or low pH values can make these enzymes lose their sensitivity [30,31]. pH is also a main factor in enzyme stabilities [32]. Each enzyme includes a suitable or optimal pH stability range [30,32]. Apart from temperature and pH, ionic strength can also affect the enzymatic reaction [33]. For more accurate and consistent results, each of these physical and chemical parameters must be considered and optimized accordingly [34]. ISFETs can be based on many materials as their detectors such as membranes and graphene [35]. Because of the physical and electrical properties of graphene, it can be applied as a sensing material in the structure of FETs [35]. On the other hand, there are no information on the development and modelling of ion-sensitive FETs, and their potential as ISFET has not been totally studied yet. The reaction between solution with different pH values and the surface of graphene has a notable effect on the conductivity of graphene [36]. This means that the detection mechanism of adsorbing the hydrogen ions from solution to carbon-based materials can be clarified as shown in Figure 2. In other words, based on the electron transfer between ion solutions and graphene surface, an analytical model of the reaction between buffer solution of different pH and graphene is presented.

Figure 2 illustrates the detection mechanism of solution with different pH using an ISFET device. Monolayer graphene on silicon oxide and silicon substrate with a deposited epoxy layer (Epotek 302–3 M, Epoxy Technology, Billerica, MA, USA) as an ISFET membrane is proposed. In this paper, pH of solution as a gate voltage is replicated due to the carrier injected to channel from it, and also pH as a sensing parameter () is suggested. Finally, the presented model is compared with experimental data for purposes of validation.

### Proposed model

The graphene nanoribbon channel is supposed to be completely ballistic for one-dimensional monolayer ISFETs for pH sensing since high carrier mobility has been reported from experiments on graphene [37]. A district of minimum conductance versus gate voltage as a basic constant relative to the electron charge in bulk graphite (*q*) and Planck's constant (*h*) is defined by *G*_0_ = 2*q*^2^/*h*[38]. So, the electron transportation of the graphene channel in ISFET can be obtained by the Boltzmann transport formula [38,39]:

(1) $$G=\frac{2q^{2}}{h}\int_{-\infty }^{+\infty }M\left(E\right)T\left(E\right)\left(-\frac{\text{df}}{\text{dE}}\right)\text{dE},$$

where *E* is the energy band distribution, *T*(*E*) is the average probability of electron transmission in the channel between source and drain which is equal to 1 (*T*(*E*) = 1) [38] because the ballistic channel is assumed for the ISFET device, *f* is the Fermi-Dirac distribution function, and *M*(*E*) is the number of sub-bands in the ISFET channel as a summation parameter over *k* point which is defined as

(2) $$M\left(E\right)=\frac{3\text{at}}{2l}{\left(\frac{4E}{3\text{at}}-2\beta^{2}\right)}^{\frac{1}{2}},$$

where *l* is the ISFET channel length, *t* = 2.7 eV which is the tight-binding energy for the nearest neighbor C-C atoms, and *β* is the quantized wave vector which can be written as

(3) $$\beta =\frac{2\pi }{\sqrt{3a_{c-c}}}\left(\frac{P_{i}}{N+1}-\frac{2}{3}\right),$$

where *N* is the number of dimer lines, *P*_i_ is the modulation index, and *a*_*c*−*c*_ = 1.42 Å is the distance between adjacent carbon atoms in the plan. The conductance of the nanoscale material is strongly dependent on both quantizing parameters, which depend on the number of sub-bands, and interface resistance, which is independent of the length [40]. Also, the Fermi-Dirac distribution function is inserted instead of the number of sub-bands in the ISFET channel. So, it is modified as

(4) $$\begin{array}{l} G=\frac{3q^{2}}{\mathrm{hl}}\sqrt{3\pi a^{3}t^{3}k_{B}T}\\ \;\times \left[\int_{0}^{+\infty }\frac{X^{-1/2}}{\left(1/\left(1+e^{X-\eta }\right)\right)}\text{dX}+\int_{0}^{+\infty }\frac{X^{-1/2}}{\left(1/\left(1+e^{X+\eta }\right)\right)}\text{dX}\right]. \end{array}$$

In order to simplify the conductance equation, we assumed *x* = (*E* − *E*_g_ / *k*_B_*T*) and *η* = (*E*_F_ − *E*_g_) / *k*_B_*T* as normalized Fermi energy. Consequently, the supposed conductance model of the graphene-based ISFET channel can be written as

(5) $$G=\frac{3q^{2}{\left(3\pi a^{3}t^{3}k_{B}T\right)}^{\frac{1}{2}}}{\text{hl}}\left[I_{\frac{-1}{2}}\left(\eta \right)+I_{\frac{-1}{2}}\left(-\eta \right)\right].$$

**Figure 3:**
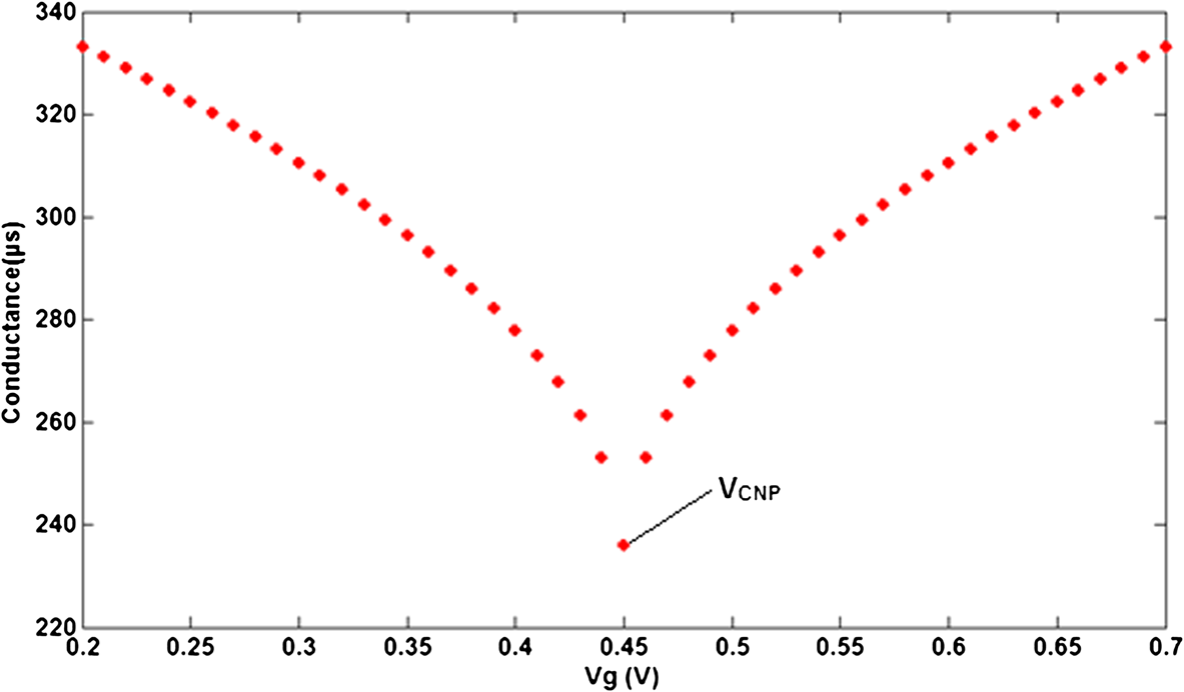
A bipolar transfer curve of the conductance model of graphene-based ISFET.

This equation can be numerically solved for different gate voltages. Thus, the proposed conductance model of the performance of the graphene-based ISFET in the nanostructured region by the conductance-voltage characteristic is evaluated in Figure 3.

By applying gate voltage between 0.2 and 0.7 V, a bipolar characteristic of FET device is monitored since the Fermi energy can be controlled by gate voltage. Based on this characteristic, it is notable that the graphene can be continuously dropped from the p-doped to the n-doped region by the controllable gate voltage. The minimum conductance is observed at the transition point between electron and hole doping. This conjunction point is called the charge-neutrality point (CNP) [41]. The conductance of the ISFET channel not only is dependent on the graphene structure and operation voltage on the source-drain channel, but also depends on the electrolyte environment and ion concentration in solution [42,43]. It has been demonstrated that different pH values can affect the ISFET conductance [42].

**Figure 4:**
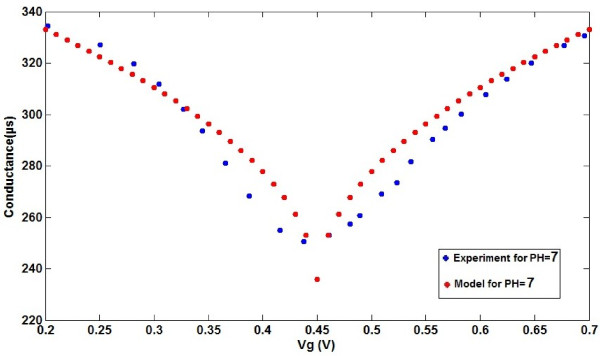
Electrical source-drain conductance versus gate voltage of graphene-based ISFET for both model and experimental data.

Before the hydrogen ion concentration was changed in the solution, a natural solution (pure water) with a buffer (pH = 7) was added in the electro-active membrane to measure the dependence of conductance versus gate voltage. There is a favorable agreement between the proposed model for pH sensing based on graphene and experimental data for non-ionic solution (pH = 7) which are extracted from [42], as can be seen in Figure 4.

**Figure 5:**
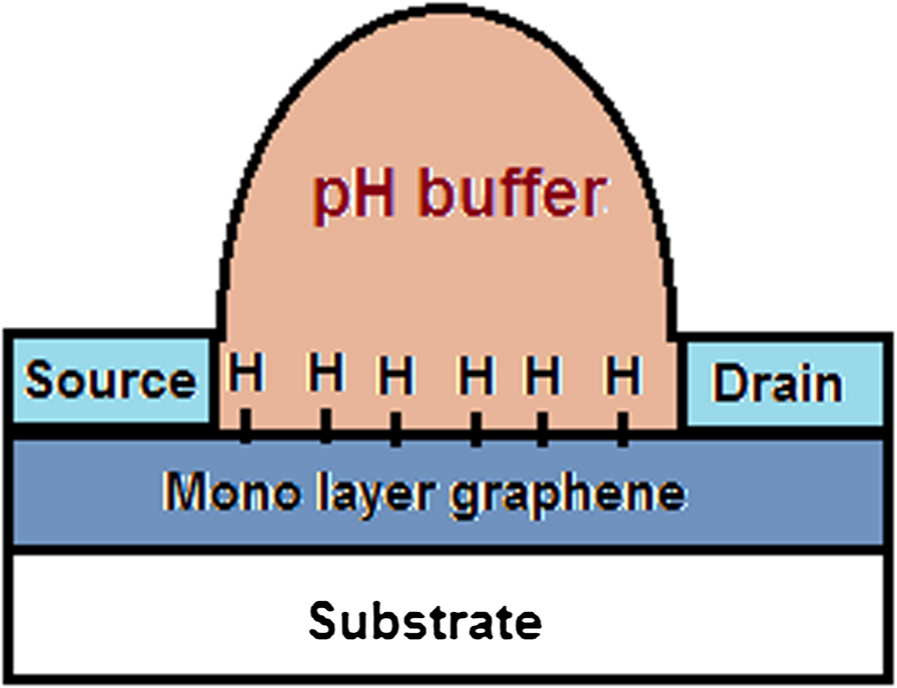
Schematic of hydrogen ion adsorption processes by surface area of single-layer graphene.

**Figure 6:**
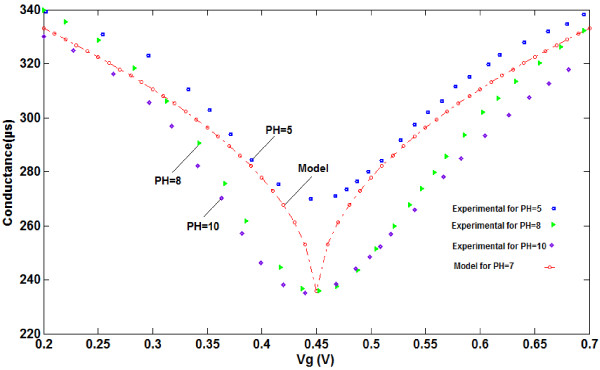
**Comparison between graphene conductance model and extracted experimental data**[42]**for different pH values.**

The conductivity of the graphene-based ISFET device is influenced by the number of carriers changing in the channel. A graphene-based ISFET with high sensitivity is applied to detect the different pH values based on conductance altering [42]. As can be seen in Figure 5, the conductance of the channel changes due to the binding of hydrogen ions in the solution to the surface of the ISFET channel. When the pH value of the solution rises from 5 to 10, less hydrogen ions will be adsorbed and the sensor will be capable of attracting less ions, leading to changes in the conductance of the graphene-based ISFET, as shown in Figure 6.

Dependent upon the source-drain conductance of the ISFET device, we can write

(6) $$G_{\text{with}\mathrm{pH}}\approx \text{pH}.$$

The focus of this paper is to present a new model for ISFET to measure pH changes; in other words, the conductance of the ISFET device as a function of different pH values is simulated and the pH factor () is suggested. Subsequently, for more understanding of the role of hydrogen ion concentration, FET modelling is employed to obtain an equation between the conductance and pH of a solution, where the suggested structure of ISFET is shown in Figure 2 with source and drain as contacts. Ultimately, different pH values can be modelled by the pH of a solution (see the following equation). This means that *G*_with pH_ can be shown as a function of pH values:

(7) $$G_{\text{with pH}}=\frac{P}{P_{H}}G_{\text{without pH}},$$

where the pH sensing factor () is assumed and *P*_H_ is the pH value. In the non-saturation region, the ISFET conductance model is shown as a function of gate voltage and the ideal conductance-voltage relation to the graphene channel of the ISFET device from Equations 5 and 7:

(8) $$G_{\text{with pH}}=\frac{P}{P_{H}}\left(\frac{3q^{2}{\left(3\pi a^{3}t^{3}k_{B}T\right)}^{\frac{1}{2}}}{\text{hl}}\left[I_{\frac{-1}{2}}\left(\eta \right)+I_{\frac{-1}{2}}\left(-\eta \right)\right]\right).$$

**Figure 7:**
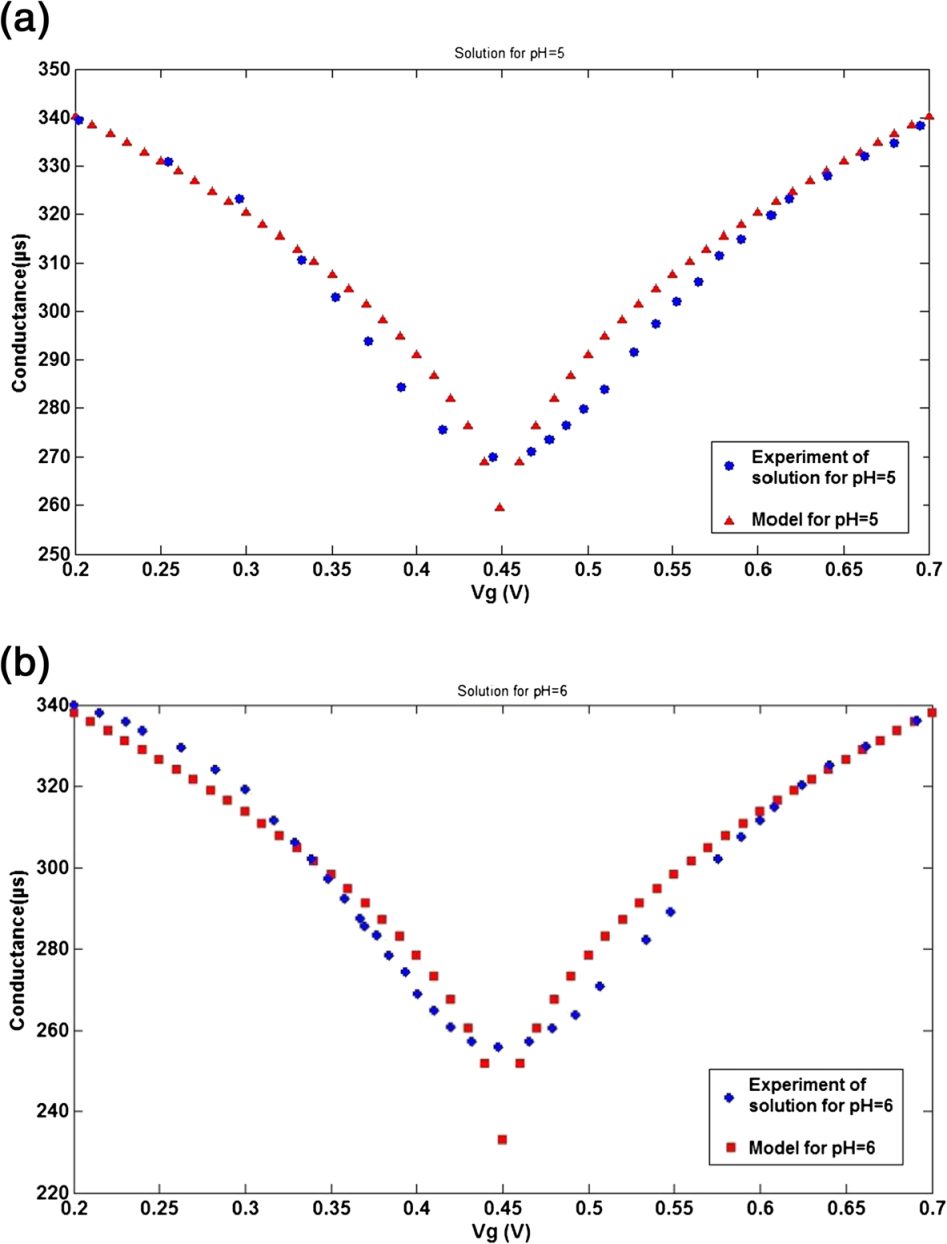
***G*-*V***_**g **_**characteristics of proposed conductance model with experimental data**[42]**.** For solutions with (**a**) pH = 5 and (**b**) pH = 6.

So, the *G*-*V*_g_ characteristics of both the model and experimental data of graphene-based ISFET for changing the pH level in solution from 6 to 7 are plotted in Figure 7.

By comparing the suggested ISFET modelling based on the proposed parameter model with experimental data in Figure 7, similar trends can be considered. In order to show all figures without overlapping, each pH value has been plotted respectively in Figure 7a,b. In addition, a detailed comparison between observed new models per pH is illustrated in Figure 7, which demonstrates acceptable agreement with experimental data. In the suggested model, different pH values is demonstrated in the form of  parameter which is in agreement with the reported data, as shown in Table 1.

**Table 1:** **Different pH values with *Ƥ *** **parameter**

***Ƥ *** **parameter values**	**pH values**
0.039105	5
0.035142	6
0.034918	7
0.034662	8
0.034437	9
0.034209	10

Therefore, based on the iteration method in Table 1, the electro-active ions absorbed by the surface of the ISFET channel as a pH sensing factor () can be suggested by the following equations:

(9) $$P=\alpha_{1}P_{H}{\;}^{\beta_{1}}$$

(10) $$P=\alpha_{2}e^{\beta_{2}\left(P_{H}\right)}\;$$

According to the saturation region of the proposed conductance model belonging to the ISFET device, Equation 11 is acceptable for both the saturation behavior and experimental data from [42]:

(11) $$P=\alpha \text{Ln}\left(P_{H}\right)+\beta$$

From extracted data, *α* and *β* parameters are calculated, where *α* = 2.7318 and *β* = 4.5044. Consequently, based on the proposed model of the ISFET device, the conductance versus gate voltage is modified as

(12) $$\begin{array}{l} G_{\text{with pH}}=\frac{\alpha \text{Ln}\left(P_{H}\right)+\beta }{P_{H}}\\ \;\left(\frac{3q^{2}{\left(3\pi a^{3}t^{3}k_{B}T\right)}^{\frac{1}{2}}}{\text{hl}}\left[I_{\frac{-1}{2}}\left(\eta \right)+I_{\frac{-1}{2}}\left(-\eta \right)\right]\right) \end{array}$$

**Figure 8:**
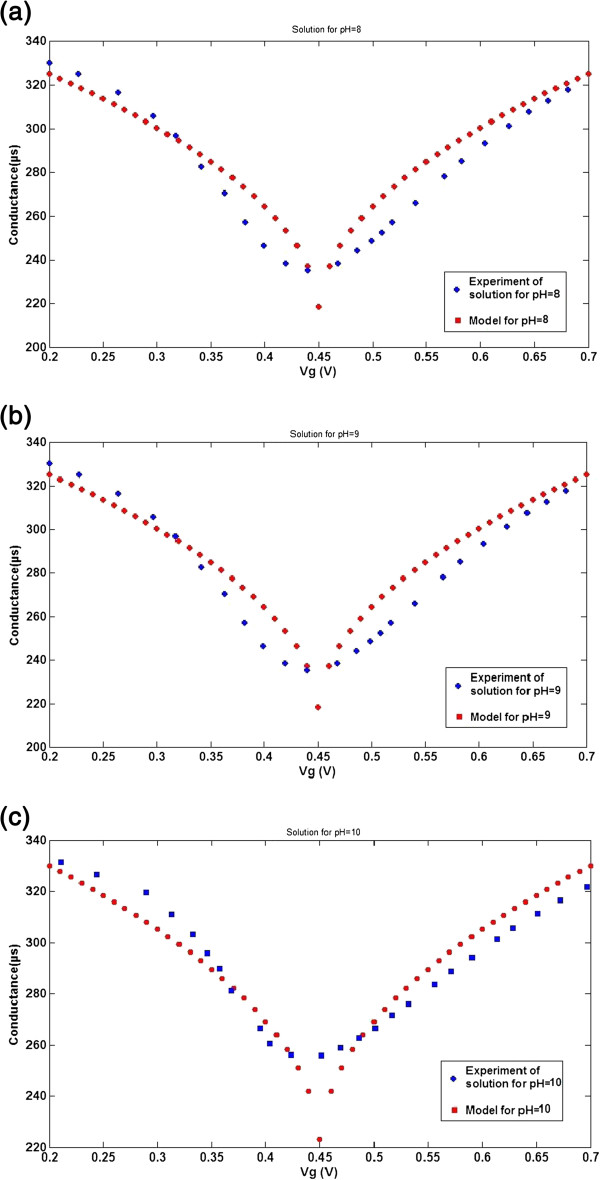
***G*-*V***_**g **_**characteristics of the proposed conductance model with experimental data.** For solutions with (**a**) pH = 8, (**b**) pH = 9, and (**c**) pH = 10.

As can be seen in Figure 8, the theoretical *G*-*V*_g_ characteristics of graphene-based ISFET for pH changes from 8 to 10 are plotted.

It is evident that the *G*-*V*_g_ characteristic curve can be controlled by the pH factor () and also the proposed model of ISFET conductance closely matches with experimental data. In both reported data and theoretical data, the decline of ISFET conductance is noticeable when the pH level increases. Also, the conductance curve is almost symmetric near *V*_CNP_, while at a large carrier concentration of about 350 to 400 μS, a saturation behavior is depicted. Comparing both experimental data and theoretical data depicted in Figure 5 reveals that when the concentration of hydrogen ions changes from pH = 7 to pH = 8, ISFET conductance decreases about 5 μS. Also, as shown in Figure 8a,b,c, each graph shows a particular value of pH. For example, when the pH value is 8, it is notable that the model is closer to the blue line (experimental data), and also in the different pH values, we can compare other ion concentrations as well. An innovative analysis of matching models using the different values in experimental data is presented in this work to verify that the conductivity of the graphene-based ISFET is moved down vertically at higher pH values. The ion-sensitive FET structure was used with monolayer graphene prepared by CVD and grown in large size on pieces of p-doped Si covered with a 300-nm substrate to measure pH changes [42]. In this study, one can claim that pH changes in the electro-active membrane will significantly and vertically shift the value of conductance in graphene (*G*_with pH_) that occurred due to ion adsorption on the surface area of the monolayer graphene sheet of the ISFET channel. Also, it is notable that the temperature remains constant (about 25°C in solution) in the suggested model as the temperature can have an effect on the behavior of the sensing parameter as well.

## Conclusions

Graphene with *sp*^2^-bonded carbon atoms has considerable potential on bio-sensing materials and electrochemical applications. The emerging potentials of nanostructured graphene-based ISFETs with high sensitivity and ability to readily detect have been applied to electrochemical catalysis through pH sensing. The conductance of an ISFET device with different pH values can be displayed by the ion concentration of the solution. In this research, the conductance of graphene is assumed as a function of pH levels (*G*_with pH_ ≈ pH), which shows the pH factor. Measurements show decreasing conductivity when the pH value of the electrolyte is increased. Especially in *V*_CNP_, the changed conductance values are clearly depicted. The suggested model verifies the reported experimental data as well. In other words, based on the good agreement between the presented analytical model and experimental data,  can be seen as a pH factor to predict graphene behavior in graphene-based ISFETs.

## Competing interests

The authors declare that they have no competing interests.

## Authors’ contributions

MJK wrote the manuscript and contributed to the analytical modelling of the presented FET via MATLAB software. Dr. FKCh and Dr. MTA revised the manuscript and coordinated between all the contributors. HKFA, MR, and AH organized the final version of the manuscript. All authors read and approved the final manuscript.
